# General population normative data for the EQ-5D-3L in the five largest European economies

**DOI:** 10.1007/s10198-021-01326-9

**Published:** 2021-06-12

**Authors:** Mathieu F. Janssen, A. Simon Pickard, James W. Shaw

**Affiliations:** 1 Department of Psychiatry, Erasmus MC, Section Medical Psychology and Psychotherapy, P.O.Box 2040, 3000 CA Rotterdam, The Netherlands; 2 Department of Pharmacy Systems, Outcomes and Policy, College of Pharmacy, University of Illinois at Chicago, Chicago, IL USA; 3 Worldwide Health Economics and Outcomes Research, Bristol-Myers Squibb, Lawrenceville, NJ USA

**Keywords:** Population norms, EQ-5D, Cost-effectiveness models, EUR5, Health

## Abstract

**Aim:**

The EQ-5D is a generic measure of health that is widely applied for health economic and non-economic purposes. Population norms can be used to facilitate the interpretation of EQ-5D data. The objective of this study was to develop a set of pooled normative EQ-5D-3L values for the five largest European economies (EUR5).

**Methods:**

EQ-5D-3L index values based on the time trade-off (TTO) were available for all EUR5 countries (n = 21,425): France, Germany, Italy, Spain, and the United Kingdom (UK). Country-specific data sets were aggregated and weighted to facilitate the derivation of norms for gender and age groups. Analyses included equal weighting and weighting by population and economy size. Norms were also calculated using the European visual analog scale-based value set (European VAS), the EQ VAS and separately by dimension.

**Results:**

Pooled mean (SD) population weighted TTO values for males/females were 0.967 (0.122)/0.959 (0.118) for ages 18–24; 0.965 (0.096)/0.954 (0.117) for ages 25–34; 0.943 (0.165)/0.936 (0.169) for ages 35–44; 0.934 (0.150)/0.921 (0.157) for ages 45–54; 0.896 (0.188)/0.875 (0.197) for ages 55–64; 0.900 (0.158)/0.839 (0.218) for ages 65–74; and 0.830 (0.234)/0.756 (0.291) for ages 75 and older. Mean values decreased and variance increased with age; females had slightly lower mean values than males across all age bands. The unequal weighting approaches produced similar point estimates with smaller variances. Mean values for the European VAS were slightly lower than those for the TTO-based index.

**Discussion:**

Normative EQ-5D-3L values can be used to benchmark the outcomes of treated patients against the health of the general population. EUR5 norms may be useful in research applications inferring to Europe or the European Union as a whole, particularly when sample size precludes analysis at the country level.

## Introduction

The EQ-5D is a generic measure of health that is applied in economic evaluations of health care interventions as well as for non-economic purposes as a health outcome measure. Applicable to a wide range of health conditions, it provides a simple descriptive profile and a visual analogue scale (EQ VAS) that can be used for assessing population health, evaluating health outcomes in clinical trials, and for routine outcomes measurement in health systems. The EQ-5D profile data can be converted into an index value (‘utility’) for health status for use in the economic evaluation of health care. Preference-based generic measures of health such as the EQ-5D and the Health Utilities Index (HUI) are recommended sources of utilities for cost-utility analysis by the Second Panel on Cost-effectiveness in Health and Medicine [1], but also by Health Technology Assessment guidelines throughout the world [2–6]. The EQ-5D is the preferred (or one of the preferred) health outcome measures recommended by pharmaceutical reimbursement authorities in 29 countries across Europe, North America, South America, Asia and Australia [2].

Due to its widespread use and evidence supporting its validity for many applications, the 3-level EQ-5D (EQ-5D-3L) is often used as the source of utilities to inform cost-effectiveness models [6–8]. Beyond this, normative data for populations and population subgroups provide baseline or reference values that can be used to benchmark interventional trial efficacy outcomes as well as to establish burden of illness as characterized in observational studies. The aim of this study was to generate a pooled set of population norms including France, Germany, Italy, Spain and the United Kingdom (UK) that represent the five largest European economies and drug markets (EUR5) [9, 10], which could facilitate communication and interpretation of results for these purposes for this important geographical and economical area. Developing a single set of population norms for the EUR5 region allows for making inferences for Europe or the European Union as a whole. Although population norms exist for these five countries separately [11], a pooled set of EUR5 norms were lacking prior to this research.

## Methods

### Data

General population-based survey data for France (N = 2892), Germany (N = 2032), Italy (N = 4709), Spain (N = 5473), and the UK (N = 6319) were identified in which measures completed by respondents included the EQ-5D-3L. Data sources included the ESEMeD (European Study of the Epidemiology of Mental Disorders) dataset (France, Italy and Spain), the Wort und Bild Verlag survey (Germany), and the 2014 Health Survey for England, using the English population as a proxy for the UK [12–14]. For the ESEMeD study, data were collected in the years 2001–2003, by computer-assisted home interviews on a nationally representative sample of the noninstitutionalized general adult population, using a stratified probability sample design. The Wort und Bild Verlag survey was conducted in a nationally representative sample of the adult population of Germany through home interviews in 2006, using ‘random-route’ sampling methods. For the Health Survey for England, computer-assisted interviews on a randomly selected sample of households in England were conducted.

### Measure

EQ-5D-3L consists of a descriptive health state classifier and a self-rating of “your health today” using the EQ VAS [15]. Each of the five dimensions of health (mobility, self-care, usual activities, pain/discomfort, and anxiety/depression) have three levels of problems on each dimension (e.g., no problems, some problems, unable to perform/extreme problems). The EQ VAS asks the respondent to rate their health today on a scale from 0 to 100, where 0 is worst imaginable health and 100 is best imaginable health.

Norms were calculated for three types of summary scores. First, for each population dataset, country-specific time trade-off (TTO)-based value sets were applied to calculate index-based utility values (i.e., for each country separately we applied each country’s corresponding value set) [16–20]. Second, the “single currency” algorithm, which is based on a VAS-based European value set (European VAS) [21], was applied to generate European VAS values. This value set was constructed using VAS valuation data from 11 valuation studies in 6 countries: Finland (1), Germany (3), The Netherlands (1), Spain (3), Sweden (1) and the UK (2), and was based on a multilevel random effects model including dummy variables for any move away from full health and one or more dimensions on level 3. The European VAS value set was also used as common value set in a publication on EQ-5D population norms for 24 countries [11]. Third, norms were calculated for the EQ VAS self-rating of health, which was also completed by respondents from all five countries.

### Analysis

First, norms were calculated by age and gender for each country separately. Subsequently, country-specific data sets were aggregated and weighted per country, to derive the EUR5 norms overall, and for gender and age-based subgroups. By weighting the five country-specific index value set data, both the norm data and the preference structure of the five countries were combined into a pooled EUR5 set of TTO-based index values. We included equal weighting (i.e., *w* = 0.20) and weighting by population size and economy size using per capita Gross Domestic Product (GDP) [22]. Population weights and GDP were based on the most recently available data from each country [23–27] (Table 1). Equal and population weights were applied at the gender and age stratum level, while GDP per capita was weighed by country. Calculation of the standard deviation associated with mean values (TTO-based index values, European VAS and EQ VAS) was done as follows:

**Table 1:** Basis for weights by economy (GDP) and population

	2016 GDP (billions USD)	2016 GDP Per capita	2016 population
France	2466.5	$38,178	66,730,453
Germany	3479.2	$42,177	82,175,684
Italy	1850.7	$30,507	60,665,551
Spain	1232.6	$26,565	46,440,099
United Kingdom	2629.2	$40,050	65,382,556

$$SD= \sqrt{\frac{\sum_{i=1}^{c}\left({n}_{i}-1\right){s}_{i}^{2}+\sum_{i=1}^{c}{{n}_{i}({\overline{x} }_{i}-\overline{x })}^{2}}{{N}_{{\text{total}}}-1}}$$

where SD is the pooled standard deviation, *n* is the sample size, *s*^2^ the variance, $\overline{x }$ the mean, and *c* the number of countries.

Additionally, results from the separate EQ-5D dimensions were pooled, applying the same weighting approaches.

## Results

A total of 21,425 respondents provided full EQ-5D-3L responses from population surveys in the EUR5 countries (Table 2).

**Table 2:** Total sample by EUR5 country and overall, by age group and gender

Age	18–24	25–34	35–44	45–54	55–64	65–74	75+	Total
Total	1920	3703	4261	3856	3075	2857	1753	21,425
Males	934	1671	1946	1777	1442	1304	741	9815
Females	986	2032	2315	2079	1633	1553	1012	11,610
France								
Total	233	510	655	610	395	319	170	2892
Males	111	233	319	286	185	127	67	1328
Females	122	277	336	324	210	192	103	1564
Germany								
Total	207	351	417	402	315	224	116	2032
Males	117	158	207	176	152	82	47	939
Females	90	193	210	226	163	142	69	1093
Italy								
Total	425	901	955	874	715	520	319	4709
Males	201	454	472	434	352	273	133	2319
Females	224	447	483	440	363	247	186	2390
Spain								
Total	568	999	1050	719	686	867	584	5473
Males	281	452	442	314	303	382	247	2421
Females	287	547	608	405	383	485	337	3052
United Kingdom (England)								
Total	487	942	1184	1251	964	927	564	6319
Males	224	374	506	567	450	440	247	2808
Females	263	568	678	684	514	487	317	3511

**Fig. 1:**
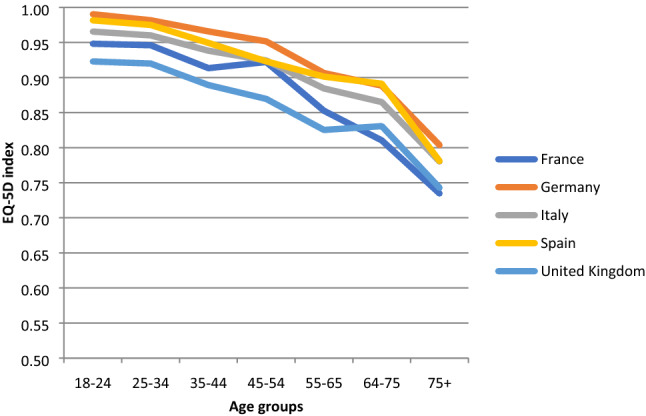
Norms for EQ-5D index (TTO-based) by age across countries

**Fig. 2:**
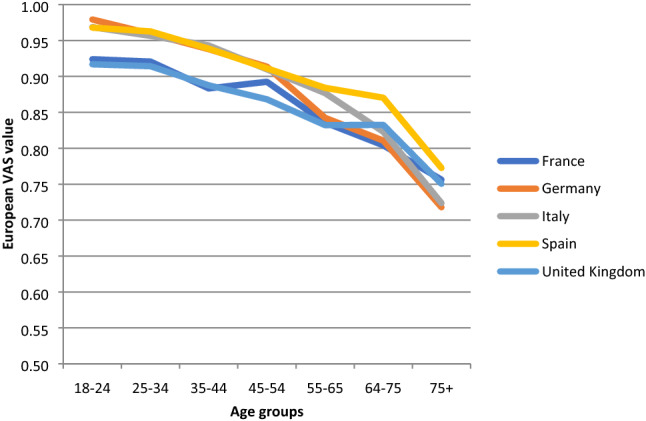
Norms for European VAS values by age across countries

**Fig. 3:**
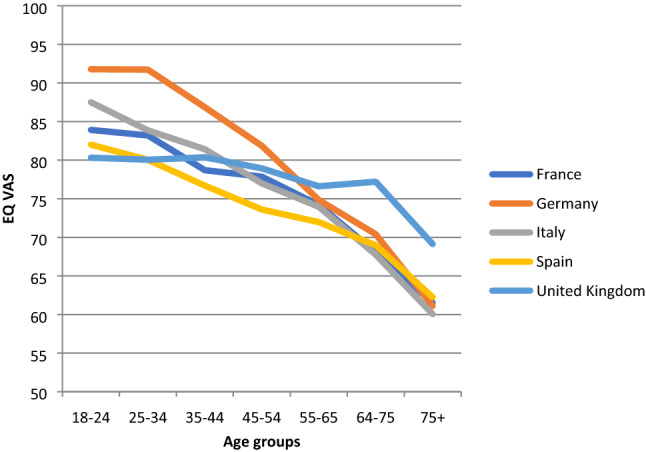
Norms for EQ VAS scores by age across countries

When stratified by age group and country, mean TTO values tended to decline as age increased in all countries (Fig. 1). A similar pattern was observed across age groups using the European VAS value set (Fig. 2). Mean self-reported EQ VAS scores declined steadily across age groups similar to the TTO and European VAS values, with the exception of the United Kingdom, where a substantial decline was only observed in the oldest age group (Fig. 3).

Overall, the mean values for each weighting approach were very similar overall and across age and gender groups (Table 3, Appendix 1: Table 5 and Appendix 2: Table 6). Pooled mean values decreased and variance increased with age when using equal weighting of countries’ values to derive norms (Appendix 1). The unequal weighting approaches produced similar point estimates with smaller variances. The means derived using these approaches most closely reflected values for Germany, which has a larger population and economy than the other EUR5 countries. Mean values for the European VAS were slightly lower than those for the TTO-based index, though a similar pattern of means across demographic strata was observed.

**Table 3:** EUR5 norm values for EQ-5D index (TTO-based), European VAS value, and EQ VAS (population weights)

		Age Groups							Total
		18–24	25–34	35–44	45–54	55–64	65–74	75 +	
EQ-5D index									
Overall	Mean	0.963	0.959	0.939	0.927	0.885	0.865	0.785	0.916
	SD	0.121	0.107	0.167	0.153	0.193	0.197	0.274	0.171
Males	Mean	0.967	0.965	0.943	0.934	0.896	0.900	0.830	0.93
	SD	0.122	0.096	0.165	0.15	0.188	0.158	0.234	0.154
Females	Mean	0.959	0.954	0.936	0.921	0.875	0.839	0.756	0.903
	SD	0.118	0.117	0.169	0.157	0.197	0.218	0.291	0.184
European VAS value									
Overall	Mean	0.949	0.941	0.919	0.900	0.852	0.824	0.740	0.890
	SD	0.129	0.121	0.157	0.156	0.184	0.191	0.243	0.171
Males	Mean	0.955	0.951	0.925	0.911	0.865	0.862	0.781	0.907
	SD	0.128	0.111	0.155	0.151	0.179	0.164	0.215	0.156
Females	Mean	0.944	0.933	0.913	0.89	0.839	0.797	0.714	0.874
	SD	0.13	0.131	0.158	0.16	0.188	0.203	0.254	0.182
EQ VAS (self-rated)									
Overall	Mean	85.4	84.4	81.1	78.4	74.5	70.6	62.5	78.3
	SD	18.3	17.6	19	19.3	19.7	21.2	25.9	20.4
Males	Mean	86.1	85.4	81.3	78.9	75.1	73	64.5	79.6
	SD	18.7	17.2	18.9	18.5	20	19.3	23.5	19.4
Females	Mean	84.6	83.6	80.9	77.9	73.8	68.8	61.2	77.1
	SD	17.5	17.9	18.9	19.9	19.1	22	26.8	21.1

**Fig. 4:**
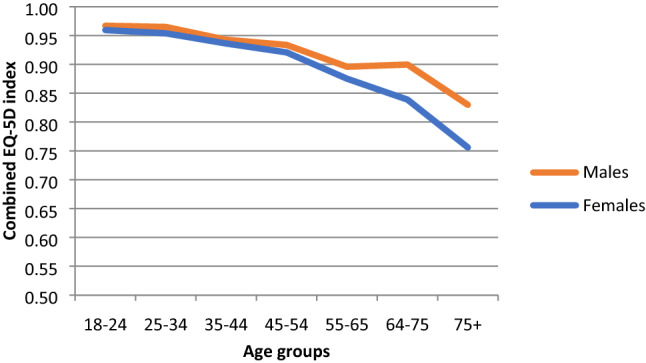
Norms for EQ-5D index (TTO-based) by age and gender (population weights)

**Fig. 5:**
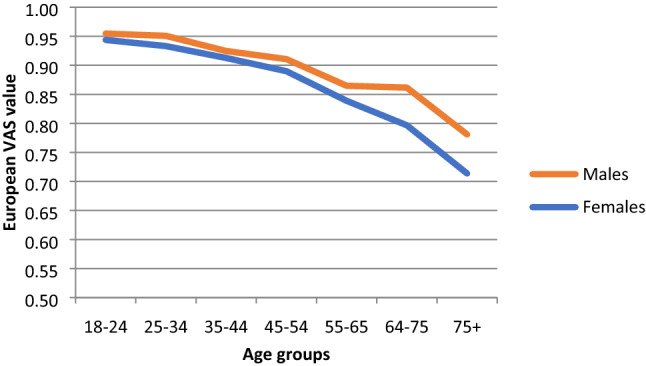
Norms for European VAS values by age and gender (population weights)

**Fig. 6:**
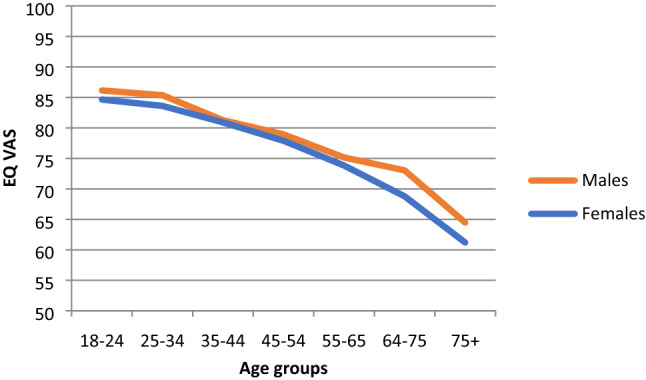
Norms for EQ VAS scores by age and gender (population weights)

Females had slightly lower mean values than males across all age bands (Table 3; Figs. 4, 5, 6). While females tended to show steady declines in TTO and European VAS value set means across age groups, mean values for males did not decline across the 55–64 and 65–74 age groups (Figs. 4 and 5).

Pooled results by dimension show that overall most problems were reported in pain/discomfort (28%), followed by anxiety/depression (13%) (Table 4). An age gradient was present across all dimensions except anxiety/depression. Self-reported pain/discomfort gradually increased across all age groups from 11 to 53%. Problems with mobility and usual activities were most notably increasing across the 55–64, 65–74 and 75+ age groups. The age gradient in these dimensions was more pronounced in females, leading up to 60% reporting any pain/discomfort, 53% problems with mobility and 43% problems with usual activities in the 75+ age group. Most reported problems with anxiety/depression occurred in females across the 55–64 and 65–74 age groups (both 19%).

**Table 4:**
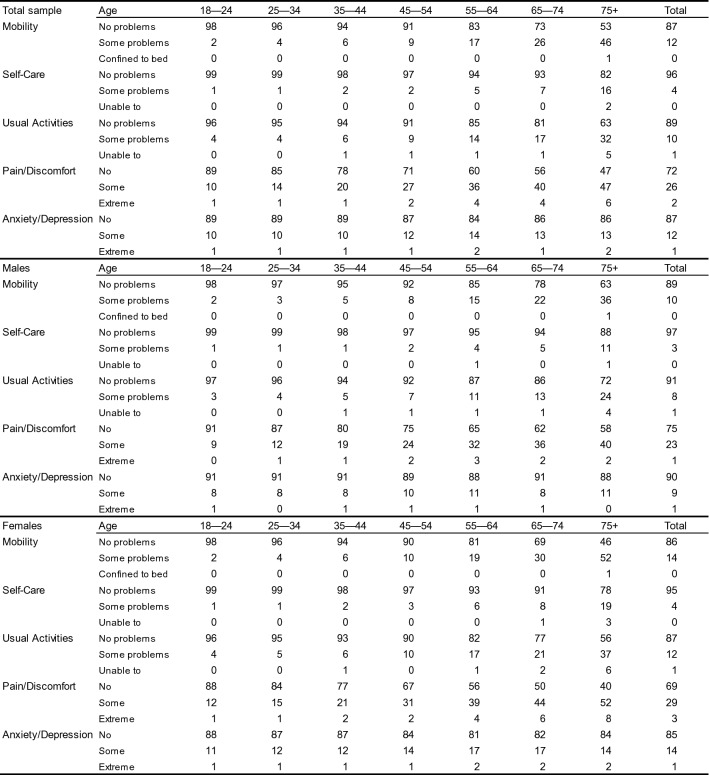
EUR5 norm values by dimension (proportions, by population weights)

## Discussion

Normative EQ-5D-3L values can be used to benchmark the outcomes of treated patients against the health of the general population. EUR5 norms may be useful in research applications inferring to Europe or the European Union as a whole, particularly when sample size precludes analysis at the country level. Population norms representing the EUR5 could facilitate a broader approach to identify unmet need based on clinical efficacy outcomes and establish burden of illness in Europe. For these purposes we recommend the population weighted set of norm values (Table 3), as weighting by population provides a better reflection of the overall EUR5 demographic composition. An additional benefit was that this weighting approach resulted in smaller variances when compared to the equal weighting approach. For purposes related to economic evaluation, we recommend the TTO-based index values, while the European VAS values may be used for non-economic studies or analyses [28]. The EQ VAS scores allow for further determining unmet need or burden of illness based on the respondents’ assessment of their overall health. Including results separately by dimension scores allows for a more detailed analysis of health complaints and symptoms across relevant subgroups, e.g., when comparing patients with increasing disease severity with the reference population. This may further help identifying relevant health aspects of unmet need across different patient populations and subgroups.

Whereas European VAS values and combined TTO values showed resemblance in our results, the self-reported EQ VAS scores typically encompassed a wider range across the scale and were overall somewhat lower, which is in line with other studies on population health measured by EQ-5D [29, 30]. For males there was a plateau effect across 55 and 75 years of age that was not present in females for which we have no current explanation. EQ VAS scores generally showed a declining age gradient, with the UK results being somewhat of an outlier with a less pronounced effect, and showing higher scores for people between 55 and 75 years of age, which might be due to a cohort effect as the UK data were the most recent.

The European Organization for Research and Treatment of Cancer (EORTC) group recently reported normative scores across 14 European countries, Canada and US to facilitate interpretation of scores in cancer [31, 32]. The EQ-5D has also been applied to facilitate interpretation of scores and relative burden of disease in cancer [33], but is much more broadly applied. Publications have previously focused on utilities for a vast array of diseases based on ICD-9 scores [34]. Catalogues of health utilities have emerged as useful resources, such as the ScHARRHUD based in Sheffield University, which focuses on generic preference-based measures such as the EQ-5D [35]. Normative scores and values for population age- and gender-specific subgroups can facilitate the interpretation of outcome results that reflect disease burden. When not available for certain countries, the availability of normative scores and values for similar countries (in terms of e.g. demographics, geography, language, infrastructure, or health care system) can be applied [36]. Care should be taken when generalizing to countries that might be (too) dissimilar to the country or region of interest, such as perhaps smaller European countries compared to the current set of EUR5 norms.

This study has several strengths and limitations. The data used in this study were based on studies conducted at different time periods. Although each population-based study was rigorously designed and collected data elements that enabled the generation of normative data by age and gender, population health in certain countries might have changed over time, as well as preferences that are reflected in the value sets. Several approaches to assigning country-specific weights were used, but the results were similar whether equal weights were applied or not, i.e., based on size of population and economy, which is indicative of a robust pooling approach for our purposes. Note that variance was smaller with unequal weighting approaches, which is due to the fact that we applied weights at the country level [37]. Considering potential changes in population health in the EUR5 countries, it could be considered to perform an update of the current pooled norm set in the future, where the EQ-5D-5L instrument might be the preferred instrument. Note that there were neither population norm data nor value sets available for the EQ-5D-5L for the EUR5 countries when this study was conducted.

A complicating issue in estimating normative values for the EUR5 countries is how Brexit will affect the UK and the European Union as economic entities. However, as the results show, trends in normative values are similar across countries and are primarily driven by age and gender. Note that the UK was still part of the European Union at the time this study was conducted and at the time of data collection. It might well be that the EUR5 ‘construct’ will continue to be useful for social, economicor other purposes in the foreseeable future.

In summary, normative EQ-5D-3L values were derived that can be used to benchmark the outcomes of patients against the health of the general population. EUR5 norms were generated for utilities based on country-specific TTO value sets, for a European-based value set estimated from VAS values, and for self-reported VAS scores. These normative values may be useful in research applications inferring to Europe or the European Union as a whole, particularly when sample size precludes analysis at the country level.

## Appendix 1

See Table 5.

**Table 5:** EUR5 norm values for EQ-5D index (TTO-based), European VAS value, and EQ VAS for the EUR5 (equal weights)

		Age Groups							Total
		18–24	25–34	35–44	45–54	55–64	65–74	75 +	
EQ-5D index									
Overall	Mean	0.966	0.960	0.938	0.924	0.884	0.865	0.780	0.915
	SD	0.119	0.115	0.186	0.179	0.217	0.223	0.284	0.193
Males	Mean	0.969	0.965	0.942	0.931	0.895	0.901	0.827	0.930
	SD	0.119	0.100	0.180	0.174	0.211	0.179	0.246	0.173
Females	Mean	0.962	0.955	0.935	0.918	0.874	0.838	0.750	0.902
	SD	0.117	0.128	0.191	0.184	0.221	0.244	0.299	0.210
European VAS value									
Overall	Mean	0.951	0.943	0.918	0.899	0.854	0.828	0.744	0.890
	SD	0.130	0.131	0.172	0.178	0.204	0.215	0.247	0.191
Males	Mean	0.956	0.951	0.924	0.910	0.868	0.866	0.787	0.908
	SD	0.126	0.118	0.167	0.171	0.199	0.185	0.222	0.172
Females	Mean	0.946	0.935	0.912	0.889	0.841	0.800	0.717	0.874
	SD	0.132	0.143	0.177	0.183	0.208	0.227	0.255	0.205
EQ VAS (self-rated)									
Overall	Mean	85.1	83.8	80.8	77.9	74.3	70.5	62.8	77.9
	SD	19.3	20.0	20.9	22.4	22.5	24.6	26.8	23.3
Males	Mean	85.8	84.7	81.0	78.3	75.0	73.0	64.8	79.2
	SD	19.2	19.0	20.5	21.5	22.8	22.6	24.5	21.9
Females	Mean	84.3	83.0	80.6	77.5	73.6	68.6	61.5	76.6
	SD	19.2	20.9	21.2	23.1	22.0	25.5	27.6	24.4

## Appendix 2

See Table 6.

**Table 6:** EUR5 norm values for EQ-5D index (TTO-based), European VAS value, and EQ VAS (GDP weights)

		Age Groups							Total
		18–24	25–34	35–44	45–54	55–64	65–74	75+	
EQ-5D index									
Overall	Mean	0.964	0.958	0.936	0.923	0.881	0.862	0.778	0.913
	SD	0.120	0.109	0.167	0.158	0.200	0.202	0.283	0.175
Males	Mean	0.967	0.964	0.939	0.929	0.892	0.897	0.822	0.928
	SD	0.122	0.098	0.165	0.155	0.194	0.162	0.246	0.158
Females	Mean	0.960	0.953	0.933	0.917	0.870	0.835	0.749	0.900
	SD	0.116	0.118	0.169	0.161	0.205	0.222	0.297	0.188
European VAS value									
Overall	Mean	0.950	0.941	0.916	0.898	0.851	0.825	0.743	0.888
	SD	0.129	0.122	0.157	0.158	0.187	0.191	0.244	0.172
Males	Mean	0.955	0.950	0.921	0.908	0.864	0.862	0.783	0.906
	SD	0.128	0.112	0.155	0.154	0.182	0.165	0.218	0.158
Females	Mean	0.944	0.933	0.910	0.888	0.838	0.798	0.717	0.873
	SD	0.129	0.131	0.158	0.161	0.191	0.203	0.254	0.182
EQ VAS (self-rated)									
Overall	Mean	85.3	84.2	81.2	78.3	74.5	70.8	63.0	78.2
	SD	18.5	17.8	18.7	19.6	19.9	21.3	26.2	20.5
Males	Mean	86.0	85	81.3	78.7	75.2	73.2	64.9	79.4
	SD	19.0	17.4	18.5	18.8	20.2	19.4	23.8	19.5
Females	Mean	84.6	83.4	81.1	77.9	73.9	69.0	61.8	77.0
	SD	17.8	18.1	18.7	20.2	19.4	22.2	27.1	21.2
